# Royal Victoria Hospital, Belfast

**Published:** 1903-12-26

**Authors:** 


### ROYAL VICTORIA HOSPITAL, BELFAST.

### ITS CONSTRUCTION AND SYSTEM OF VENTILATION.

Before the Royal Institute of British Architects, on the 14th inst., papers were read on the Royal Victoria Hospital, Belfast, by Mr. William Henman, the architect, and Mr. Henry Lea, the engineer. There was a large attendance. Mr. Aston Webb, R.A., F.S.A., the President of the Inssitute, took the chair.

Mr. Henman said there was much that was unique in its inception, original in its design, and novel in its equipment, which had attracted attention to the Royal Victoria Hospital, Belfast. It seemed, therefore, advisable that an authentic description should be published of the reasons which had led to what had been termed a "revolution in hospital design," as well as of the methods and means by which it had been accomplished. If the plan was revolutionary in its tendency, he hoped to show that it was a serious endeavour to simplify, and, if possible, to improve hospital design. Whether it was destined to secure permanent approval, or be pointed at as an example to be avoided, depended upon, first, whether the essentials in hospital design had been properly appreciated and applied; secondly, whether those having the care and management of the institution would maintain it in efficiency. As regards the development of the design in question, distinct variations in hospital planning had been principally brought about as knowledge increased of the necessity for efficient ventilation and of the means by which it could be secured. Hence, with mechanical means at their disposal, an attempt was made at Belfast to design a hospital that could be efficiently ventilated by such means. The author acknowledged his indebtedness to Mr. William Key, of Glasgow, who had proved the possibility of securing efficient ventilation on the plenum system—the system which had been adopted at the hospital.

The wards of a hospital are occupied both day and night by patients in a low state of health, and continuous change of air, with an equable temperature and freedom from draughts, secured without noise or dirt, is necessary to the cure of the patients. The question therefore is, How can hospital wards be designed and arranged so as economically and effectively to secure the desired conditions? Reference was made to correspondence which had appeared in the *Builder* suggesting that in consequence of the proved success of plenum ventilation, combined with antiseptic treatment, it might be possible to dispense with the "pavilion" arrangement of wards, and advocating double wards divided longitudinally by dwarf partitions. This idea had seemed to the author altogether wrong, and he had set forth his views in a letter in the *Builder* of August 8, 1896. Here he suggested that, "instead of erecting detached pavilions of several stories, it might be better to spread out the wards on one story only, placed side by side and lighted by continuous lantern lights. Such an arrangement would secure greater comfort to the patients, simplify ventilation by mechanical means, and very considerably reduce corridor communication as well as dispense with the inconvenience of staircases and lifts, thereby facilitating administration. For the accommodation of the staff there would be no objection to buildings of several stories, but with all the patients compactly arranged on one floor level their wants could be easily supplied, and other difficulties of the pavilion plan would be overcome." The letter concluded: "Only those who study what is possible with the plenum system of ventilation properly applied can realise the practicability of such an arrangement; yet by its employment I feel convinced that some such revolution in hospital planning will be accomplished, and do not doubt that in time it will be demanded, partly in consequence of the great cost of the pavilion plan, but more particularly in consequence of the excessive labour thereby involved."

When the author was requested to meet the Committee of the Royal Victoria Hospital, Belfast, reference was made to the suggestions contained in the above letter, and he was questioned as to the practicability of constructing a hospital such as he had proposed. On attempting to fit together, on entirely new lines, the intricate requirements of a complete hospital for 300 patients and a large staff, he began to realise the difficulties he had imposed upon himself. With the assistance of his partner, Mr. Thomas Cooper, the plan in time, however, assumed the generally symmetrical arrangement in which it now appeared in the erected building.

The author referred to the opposition raised against the scheme before it was finally sanctioned by the committee. Professor Byers, Member of Council of the British Medical Association, and honorary member of the medical staff, averred that, "when first he heard that it was proposed to place all the wards side by side without intervening open spaces, to light them principally from above, and to have no windows to open, it appeared to him so contrary to all his preconceived ideas on hospital design that he determined to oppose the carrying out of such a plan by every legitimate means, and, to enable him to do so effectually, he set about independently to study the subject in all its bearings; but, to his surprise, the more thoroughly he probed it the more and more convinced he became that Mr. Henman was right." The cost of the buildings, including all engineering requirements and a complete steam laundry, was but a trifle over £300 per patient's bed—proof that the arrangement of plan is capable of being carried out at an economical figure. The site is six acres in extent, to which another six is to be added. The hospital stands comparatively high, has a pleasant outlook in every direction, and is readily accessible from most of the large manufactories and works, and from the poorer parts of the city, whence the majority of the patients will come. From west to east there is a fall in the level of the ground of over 20 feet, of which advantage has been taken; by keeping the main floor level well above the ground adequate height is secured for the principal fresh air duct, which runs under the main corridor, for the branch ducts conveying fresh air to the several wards and accessory rooms, also for a pipe-duct running parallel with the principal air duct—a necessary provision, so that heat from the steam and hot water pipes may not penetrate the buildings during the summer months, and that convenient access may be obtained to all piping.

Opening directly from the hall opposite the porch entrance runs the main corridor from east to west, some 450 feet long and 9 feet wide. Branching southward are 17 short corridors, giving access to as many wards, each with 14 beds, with their accessory rooms, all practically under one roof. The eight wards to the east are for medical cases; then come eight wards for surgical cases, and one for gynæcological cases. To the north are two for ophthalmic cases.

The author described the general arrangement of the rooms—male and female wards, class rooms, clinical rooms, operating rooms, ward kitchens, store rooms for clothing, nurses' and cleaners' rooms, sanitary conveniences, etc.; also the "Extern," or out-patients' department, with its waiting-hall and medical, surgical, and specialists' consulting-rooms, examination rooms, dispensary, etc. Intercepting lobbies between wards and conveniences are dispensed with. Open windows with plenum ventilation are objectionable, and without open windows intercepting lobbies are an anomaly. By simple adjustment, air from sanitary turrets is prevented from entering the wards, air pressure in their direction being maintained from the wards outwards. To the ward kitchens no doors are provided. With plenum ventilation an equable temperature and freedom from draught are secured, consequently inner doors are required only for the sake of privacy, or where difference of temperature is desired to be maintained.

Two small detached buildings at the west end of the site are for isolation purposes. They receive fresh air by a continuation of the main duct underground, and, although at least 600 feet away from the fans, are amply supplied with fresh air.

The author showed sections through a portion of the ward-block to give a general idea of the method of lighting and of the construction adopted. The windows are not skylights as generally understood, but may be more properly defined as clerestory windows, slightly sloping, and glazed with half-inch plate glass. The result is most perfect lighting to every portion of the building. Surgeons using the operating-rooms state that nothing could be better for their purpose; the light is ample, well-diffused, and free from shadowing. An advantage of plenum ventilation is that the cubical contents of buildings may be very considerably reduced. Given sufficient floor area for nursing and teaching purposes, the height of wards need be no more than appearance demands, and when lighted, as they are in this instance, the cubical contents are much reduced, being not more than two-thirds of what is ordinarily required.

After referring to the administrative buildings, the author described the various fittings and appointments of water-closets, sinks, bath-rooms, operating-rooms, post-mortem rooms, pathological and microscope rooms. The whole of the sanitary appliances were carried out from the architects' designs by Messrs. Morrison and Ingram, and fixed by Mr. John Dowling, plumber, of Belfast.

Mr. Henman concluded with a word of caution about plenum ventilation. It is essential it should be applied with full knowledge and by those competent to deal effectively with it. Distrust an engineer who will give a scheme for ventilating any building indiscriminately on the "plenum" or "extraction" system, or by what are termed natural means; but try to realise that every building should be designed for the particular method of ventilation intended to be employed, and that the means for procuring ventilation must be specially designed on scientific principles to meet the actual requirements of the building.

Mr. Henry Lea, M.Inst C.E., who followed with a paper on the "Engineering Work of the Belfast Hospital," speaking of the ventilation, said that the design which Mr. Henman had originated facilitated to an extraordinary degree a simple arrangement of air-ducts of ample proportions. To emphasise the importance of this point, he mentioned two large buildings now ventilated by mechanical means, each having about 13,000,000 cubic feet of fresh air driven through them per hour. In one building, owing to the liberality with which the air-ducts are proportioned, the total amount of power required to drive the fans was 19 indicated horse-power; in the other building, owing to the air-ducts being restricted and very crooked, the power required was 53 indicated horse-power. Putting this into money value, in one case the driving power cost £766 per annum, and in the other £2,164 per annum.

Another point in relation to economical working is that the fans are driven by a steam engine, the exhaust steam from which is utilised for heating the water for the baths and lavatories. The lecturer showed that there was a saving under this head of £275 per annum.

The main engineering features in connection with the ventilation of the hospital having been fully detailed, the lecturer treated of the hot and cold water supply, sterilizing apparatus for the operating-rooms, laundry arrangements, and electric light system.

The lectures were illustrated by limelight views of the hospital buildings and wards and by plans of the hospital.

Sir John Holder, chairman of the Birmingham General Hospital, proposed a vote of thanks to Messrs. Henman and Lea. The Birmingham Hospital, he said, was designed and built by Mr. Henman, who had every reason to be proud of his work. That building was designed for the natural system of ventilation, but they were recommended to adopt the plenum system, and after visiting the hospital at Glasgow in which it was in use they decided in its favour, and were very pleased they had done so. They were able to keep a very even temperature and a constant supply of fresh air. He had seen the Johns Hopkins Hospital, the Guild Hospital at Montreal, and other famous institutions, but was more than satisfied with his own.

Dr. Christopher Childs said that, though not an expert, he had given some time to the consideration of the plenum system, but though convinced it was the best system for ventilating crowded buildings like churches, schools, and theatres, he questioned whether it was the best for hospitals, and as a physician he must throw in a discordant note. They had air analysed for impurities and for bacteria, but the distinctive quality of fresh air they knew very little about. Air on the plenum system did not contain that invigorating essence they all recognised as the peculiar property of fresh air. It might be due to the rapidity of movement, but there was no doubt as to its wonderful effects, especially as shown in the open-air treatment of consumption.

Mr. Alfred Saxon Snell asked that, in view of the lateness of the hour—it was by his time past 10 o'clock—and the importance of the questions raised, that the discussion should be adjourned, and this was seconded by Mr. Hare.

